# The influence of HIV infection and antiretroviral treatment on pulmonary function in individuals in an urban setting in sub-Saharan Africa

**DOI:** 10.4102/sajhivmed.v22i1.1312

**Published:** 2021-11-15

**Authors:** Oda E. van den Berg, Erica J. Shaddock, Sarah L. Stacey, Charles Feldman, Roos E. Barth, Diederick E. Grobbee, Willem D.F. Venter, Kerstin Klipstein-Grobusch, Alinda G. Vos

**Affiliations:** 1 Julius Center for Health Sciences and Primary Care, University Medical Center Utrecht, Utrecht, the Netherlands; 2 Charlotte Maxeke Johannesburg Academic Hospital, Division of Pulmonology and Critical Care, School of Clinical Medicine, Faculty of Health Sciences, University of the Witwatersrand, Johannesburg, South Africa; 3 Department of Internal Medicine and Infectious Diseases, University Medical Center Utrecht, Utrecht, the Netherlands; 4 Ezintsha, University of the Witwatersrand, Johannesburg, South Africa; 5 Julius Global Health, Julius Center for Health Sciences and Primary Care, University Medical Center Utrecht, Utrecht, the Netherlands

**Keywords:** HIV, antiretroviral therapy, tuberculosis, spirometry, sub-Saharan Africa, obstructive lung disorder, COPD, asthma, pulmonary function

## Abstract

**Background:**

With the roll-out of antiretroviral treatment (ART), the life expectancy of people with HIV and, hence, morbidity from non-communicable diseases, including pulmonary diseases, have increased.

**Objectives:**

This research study aims to investigate whether HIV infection and ART use are associated with pulmonary function, given the high frequency of pulmonary infections, including tuberculosis (TB), associated with HIV.

**Method:**

Adults living with HIV (ART-naïve, on first- or second-line ART), and age and sex matched HIV-negative controls were included in a cross-sectional study in Johannesburg, South Africa. Spirometry was performed to determine lung function, measuring the forced expiratory volume in one second (FEV1), the forced vital capacity (FVC) and the FEV1/FVC ratio before (pre), and after (post), short-acting bronchodilator. The association of HIV infection and ART use with pulmonary function was analysed using linear regression models, adjusting for age, gender, body surface area (BSA), employment, education, smoking and TB.

**Results:**

Overall, 548 participants (62% women) were included with a mean age of 38 (standard deviation [s.d.] 9.5) years. No effect of HIV or ART on post-FEV1 was observed in adjusted analysis. Additional adjustment for TB resulted in a higher post-FEV1 in participants on ART compared with HIV-negative participants, whereas TB was associated with a lower FEV1. No effect of HIV and ART on post-FEV1/FVC was observed.

**Conclusion:**

HIV infection and ART use were not associated with reduced pulmonary function in this urban African population. Tuberculosis showed a mediating effect on the association between HIV, ART and pulmonary function.

## Introduction

Approximately 70% of all people with HIV infection live in sub-Saharan Africa (SSA), with South Africa constituting the largest HIV epidemic profile in the world.^1^ Before the roll-out of antiretroviral therapy (ART) programmes, the most frequent complications of HIV infection included pulmonary infections, which were a major cause of morbidity and mortality rates.^2^ The widespread use of ART for the treatment of HIV has contributed to an increased life expectancy among people living with HIV (PLHIV), which is subsequently associated with increases in non-communicable diseases in PLHIV.^2,3^ Among non-communicable diseases, obstructive lung disorders (OLDs) constitute a major class.^4^ The burden of chronic respiratory diseases is generally increasing across the globe, and asthma and chronic obstructive pulmonary disease (COPD) are among the main causes of mortality and morbidity.^5^ Whilst risk factors, such as smoking and opportunistic infections, contribute to this increased risk, the literature suggests that HIV infection and ART use may be associated with the development of OLDs.^6,7^ Viral suppression resulting from ART could contribute to the preservation of lung function.^7^ However, some studies suggest that there is a direct negative effect of ART on pulmonary function.^8,9,10^ Potential explanations include a direct toxic effect of ART on the lungs, a renewed response to subclinical pulmonary infections because of the restoration of the immune system and/or the development of autoimmunity. In South Africa, the adult ART coverage is 73%.^1^ Patients failing first-line ART, consisting of non-nucleoside reverse-transcriptase inhibitor-based (NNRTI) regimens, are switched to second-line ART, including a protease inhibitor.^11^ To date, there have been no data on potential differential effects of distinct ART classes on lung function, and the results from studies carried out in high-income countries cannot be generalised to SSA because of differences in socio-demographic characteristics of the HIV population and the differences in the burden of infectious respiratory diseases.^3^

Tuberculosis (TB) remains a major health challenge among HIV-positive people in South Africa. Within the first year of HIV infection, the risk of active TB doubles.^12^ Tuberculosis is thought to mediate the effect of HIV and ART on OLDs, as HIV increases the susceptibility to TB, which is strongly related to adverse pulmonary function.^12,13,14^ However, only a few studies on the influence of HIV on pulmonary function have taken into account the possible contribution of TB. This study aims to investigate the influence of HIV, first-line ART and second-line ART on pulmonary function in an urban African population, taking into account the role of TB.

## Methods

A four-arm comparative, cross-sectional study was performed, including people aged ≥ 18 years between July 2016 and November 2017. The study site was located at the Charlotte Maxeke Johannesburg Academic Hospital, central Johannesburg, South Africa. The methods have been described previously.^15^ In line with the inclusion criteria of the randomised controlled trials (RCTs) from which the participants were selected, four different groups consisting of PLHIV but not yet or less than 8 weeks on ART (HIV+, ART–), PLHIV and on first-line tenofovir-containing ART regimens for at least 2.5 years (HIV+, ART1), and those on second-line ART for at least 6 months (HIV+, ART2) and HIV-negative controls (HIV–) were identified.

Three ongoing RCTs, conducted by the Wits Reproductive Health and HIV Institute (Wits RHI),^14^ were used for the recruitment of participants. The HIV+, ART– patients were recruited from an ongoing open-label RCT investigation of dolutegravir and two different prodrugs of tenofovir as first-line ART in people newly diagnosed with HIV, and were recruited from routine HIV services in local clinics in central Johannesburg.^16^ Patients were approached for participation in this study at any time from their enrolment in the RCT up to a follow-up duration of 36 weeks. None of the HIV+, ART– participants was on ART for more than 8 weeks upon enrolment in this study. Participants in the HIV+, ART1 group were recruited from a RCT completed in 2016, which investigated low-dose stavudine versus tenofovir in first-line ART regimens. These participants were recruited from the same clinic.^17^ Only participants to receive tenofovir were asked to participate in this study by phone in a random order until the required number of participants was reached.

The HIV+, ART2 group was selected from an open label randomised study comparing two second-line protease-inhibitor-based ART regimens.^18^ Participants were approached for enrolment in this study during baseline or one of the follow-up visits. The main exclusion criteria for all RCTs were impaired kidney and/or liver function, hepatitis B infection and pregnancy. For recruitment of the HIV–group, participants living with HIV were asked to refer an uninfected family member or a friend or contact with unknown HIV status of the same sex and age (±5 years) to participate in the study. All participants without a known HIV-positive status were tested for HIV on enrolment, according to national testing guidelines.^19^ If a participant tested positive for HIV without a history of ART use, they were assigned to the HIV+, ART– group and referred to a local clinic to start ART. However, if a participant tested positive for HIV and was found to be on ART already, they were assigned to the HIV+, ART1 or HIV+, ART2 group depending on their ART regimen.

Data were collected during a single visit through questionnaires, physical examination and spirometry. A modified version of the WHO STEP instrument was used to assess information on demographics, ethnicity, medical history, concomitant drug use, exposure to harmful liquids, gases or material and a family history of pulmonary disease.^20^ The respiratory questionnaire was based on the British Medical Research Council Respiratory Questionnaire,^21,22^ the ATS-DLD-78-A,^23^ the World Health Survey^24^ and questionnaires used in other publications.^25,26^ Tuberculosis was defined as self-reported history of TB or pneumonia.

Spirometry, with pre- and post-bronchodilator measurements, was performed by trained researchers using a hand-held CareFusion 2009 spirometer and Spida 5 software. The participants were seated when performing the manoeuver. In order to obtain sufficient spirometry results, three acceptable manoeuvers were needed in which the two largest values for forced expiratory volume in one second (FEV1) and forced vital capacity (FVC), respectively, were at maximum 150 mL apart. In addition, efforts were considered acceptable if the individual performed the maneuver with maximum inspiration, a good start, a smooth continuous exhalation and maximal effort, without evidence of early termination, inconsistent effort, leak or an obstructed mouthpiece. If the results were not sufficient, the measurements were repeated up to a maximum of eight times. After obtaining the first measurements, a bronchodilator was administered (400 µg salbutamol) to the participant using an aerochamber. After 15 min, post-bronchodilation spirometry was performed using the same method as described above. All calibrations and tests were performed in accordance with the American Thoracic Society (ATSIII) and European Respiratory Society recommendations,^27^ and only tests that met the above-mentioned criteria were included in the analysis. In cases where participants had only one or two reproducible post-bronchodilator flow volume curves, but these were comparable with the pre-bronchodilator flow volume curves, the post results were used for analysis. The respiratory parameters measured were as follows: FEV1, FVC and the FEV1/FVC ratio before (pre) and after (post) short-acting bronchodilator. The highest post-bronchodilator FEV1 and FVC values were used for analysis. Spirometry results that revealed the presence of disease were reviewed by a pulmonologist. The highest post-bronchodilator FEV1 and FVC values were used for analysis.

For the current study, OLDs were defined as the presence of COPD according to the lower limit of normal (LLN) cut-off and reversible airflow obstruction. As the gold standard definition of COPD, FEV1/FVC < 0.70,^28^ overestimates airflow obstruction in the elderly and underestimates airflow obstruction in the young,^29^ we chose to include an LLN to define OLDs. The LLN is defined as a FEV1/FVC ratio below the fifth percentile of the predicted value. The Global Lung Initiative equation category ‘Afro-American’ was used to define normal values.^30^ The predicted values of FEV1 percentage were used to classify COPD severity.^28^

According to the National Heart, Lung and Blood Institute, reversible airflow obstruction is present when the FEV1 increases by 12% and 200 mL after inhalation of the bronchodilator, which may indicate the presence of asthma.^31,32^

Continuous descriptive data are presented as means and standard deviations (s.d.) or, in the case of non-normality, as medians and interquartile ranges (IQR) and nominal data with frequency count (percentage [%]). The uncorrected post-bronchodilator spirometry results are presented per group.

### Data analysis

Demographic and clinical characteristics of the population were presented using mean and s.d., or median with IQR in the case of non-normality, for continuous data. Nominal data were presented using frequency counts (percentage, [%]). In Table 2, the uncorrected post-bronchodilator spirometry results were presented for the four different groups. Outcomes included the number of people with a FEV1/FVC < LLN (COPD), a FEV1/FVC < 0.70, an increase in FEV1 of 12% and 200 mL (asthma), and OLD based on the LLN.

**TABLE 1:** Characteristics of the study population.

Characteristics	HIV-negative (*n* = 149)						HIV-positive																	
							ART-naïve (*n* = 100)						First-line ART (*n* = 90)						Second-line ART (*n* = 192)					
	*n*	%	Mean	s.d.	Median	IQR	*n*	%	Mean	s.d.	Median	IQR	*n*	%	Mean	s.d.	Median	IQR	*n*	%	Mean	s.d.	Median	IQR
**Demographic characteristics**																								
Age (years)	-	-	34.7	10.6	-	-	-	-	34.1	8.4	-	-	-	-	36.9	6.4	-	-	-	-	43.1	8.1	-	-
Women	76	51.0	-	-	-	-	63	63.0	-	-	-	-	53	58.9	-	-	-	-	136	70.8	-	-	-	-
**Education**																								
Primary school or less†	12	8.1	-	-	-	-	12	12.1	-	-	-	-	10	11.2	-	-	-	-	26	13.7	-	-	-	-
More than secondary‡	63	42.6	-	-	-	-	37	37.4	-	-	-	-	30	33.7	-	-	-	-	70	36.8	-	-	-	-
Employment§	49	33.1	-	-	-	-	53	53.5	-	-	-	-	76	85.4	-	-	-	-	130	67.7	-	-	-	-
Marital status¶	31	20.9	-	-	-	-	15	15.2	-	-	-	-	35	39.3	-	-	-	-	74	38.5	-	-	-	-
**Tobacco smoking**																								
Never	89	60.1	-	-	-	-	68	68.7	-	-	-	-	71	78.9	-	-	-	-	161	83.9	-	-	-	-
Former	6	4.1	-	-	-	-	6	6.1	-	-	-	-	7	7.8	-	-	-	-	14	7.3	-	-	-	-
Current	52	35.4	-	-	-	-	25	25.3	-	-	-	-	12	13.3	-	-	-	-	17	8.9	-	-	-	-
Pack-year	-	-	-	-	3.5	1.0–8.4	-	-	-	-	3.4	1.7–5.5	-	-	-	-	1.5	0.5–4.0	-	-	-	-	7.4	2.3–12.0
Marijuana smoking, ever	18	12.2	-	-	-	-	8	8.3	12	13.5	-	-	-	-	4	2.1
**Occupational exposure**																								
Mining work > 1 year	0	0.0	-	-	-	-	1	1.0	-	-	-	-	0	0.0	-	-	-	-	2	1.1	-	-	-	-
Dusty job > 1 year	2	1.3	-	-	-	-	0	0.0	-	-	-	-	7	8.0	-	-	-	-	5	2.6	-	-	-	-
Chemicals or fumes	2	1.4	-	-	-	-	0	0.0	-	-	-	-	6	6.7	-	-	-	-	3	1.6	-	-	-	-
History of tuberculosis or pneumonia	8	5.4	-	-	-	-	10	10.2	-	-	-	-	20	22.2	-	-	-	-	99	52.1	-	-	-	-
**HIV-related characteristics**																								
Year since diagnosis	-	-	-	-	-	-	-	-	-	-	0.0	0.0–0.0	-	-	-	-	4.0	3.0–6.0	-	-	-	-	9.0	7.0–12.4
Time on ART (months)	-	-	-	-	-	-	-	-	-	-	0.0	0.0–0.0	-	-	-	-	39	35–48	-	-	-	-	96	72–126
CD4 count cells	-	-	-	-	-	-	-	-	-	-	285	194–401	-	-	-	-	416	275–581	-	-	-	-	621	422–799

Note: Outcomes are in *n* (%) or mean (s.d.) unless otherwise specified.

ART, antiretroviral therapy; IQR, interquartile range; s.d., standard deviation.

† , Includes no formal schooling, less than primary school and primary school completed;

‡ , includes matric completed and college or university completed;

§ , Employed versus no employment, retired or student;

¶ , Married or in a stable relationship versus single, divorced or widowed.

**TABLE 2:** Post-bronchodilator spirometry results.

Clinical index	All					HIV−					HIV +, ART −					HIV+, ART1					HIV+, ART2			
	*n*	%	Mean	s.d.		*n*	%	Mean	s.d.		*n*	%	Mean	s.d.		*n*	%	Mean	s.d.		*n*	%	Mean	s.d.
FEV1 (L)	-	-	2.85	0.69		-	-	3.00	0.76		-	-	2.96	0.61		-	-	2.97	0.72		-	-	2.63	0.60
FEV1% predicted†	-	-	0.99	0.15		-	-	0.98	0.15		-	-	1.00	0.12		-	-	1.00	0.15		-	-	0.98	0.16
FVC (L)	-	-	3.40	0.78		-	-	3.52	0.84		-	-	3.47	0.73		-	-	3.54	0.82		-	-	3.19	0.70
FVC% predicted†	-	-	0.97	0.14		-	-	0.96	0.13		-	-	0.98	0.12		-	-	0.99	0.14		-	-	0.97	0.15
FEV1/FVC ratio	-	-	0.84	0.07		-	-	0.85	0.07		-	-	0.85	0.06		-	-	0.84	0.06		-	-	0.82	0.07
FEV1 < 80% predicted†	49	9.2	-	-		12	8.1	-	-		4	4.1	-	-		8	9.4	-	-		25	13.2	-	-
FEV1/FVC < LLN (COPD)†	16	3.1	-	-		3	2.0	-	-		2	2.0	-	-		2	2.4	-	-		9	4.8	-	-
FEV1/FVC < 0.70	17	3.3	-	-		4	2.7	-	-		2	2.0	-	-		2	2.4	-	-		9	4.8	-	-
Asthma (ΔFEV1,12% and 200 mL)	33	6.3	-	-		5	3.4	-	-		6	6.1	-	-		5	5.9	-	-		17	9.0	-	-
OLD	44	8.3	-	-		8	5.4	-	-		8	8.2	-	-		6	7.1	-	-		22	11.6	-	-

Note: Outcomes are in *n* (%) or mean (s.d.).

HIV–, HIV-negative; HIV+, ART–, HIV-positive not yet on ART; HIV+, ART1, HIV-positive on first-line ART; HIV+, ART2, HIV-positive on second-line ART; FEV1, forced expiratory volume in 1 s; FVC, forced vital capacity; LLN, lower limit of normal; OLD, obstructive lung disorder (FEV1/FVC < LLN plus asthma); s.d., standard deviation; COPD, chronic obstructive pulmonary disease.

† , Based on GLI reference equations ‘Afro-American’.

Next, post-FEV1 and post-FEV1/FVC were compared across the groups by linear regression analysis, with the HIV-negative group as reference, as presented in Table 3. Stratification for TB was not feasible because of the small sample size within the stratum of TB-positive, HIV-negative. The first model included all groups with no adjustments; the second model was adjusted for age, sex and body surface area (BSA) calculated by Mosteller’s formula.^33^ The third model was adjusted for age, sex, BSA, smoking, employment and education. Smoking is a known confounder in the relation between HIV and pulmonary function. Employment and education were added as proxy for socio-economic status (SES). The fourth model was additionally adjusted for TB.

**TABLE 3:** HIV and antiretroviral therapy status on mean (a) post-forced expiratory volume in 1 s and (b) post-forced expiratory volume in 1 s/forced vital capacity.

Linear regression model	HIV-negative	HIV-positive					*P*
		ART-naive	*P*	First-line ART	*P*	Second-line ART	
**(a) HIV and ART status on post-FEV1**							
Model 1	Ref	−0.039 (−0.210 to 0.132)	0.651	−0.022 (−0.200 to 0.157)	0.811	−0.371 (−0.515 to −0.227)	< 0.001
Model 2	Ref	0.056 (−0.065 to 0.177)	0.367	0.103 (−0.023 to 0.229)	0.110	0.015 (−0.095 to 0.125)	0.795
Model 3	Ref	0.066 (−0.058 to 0.189)	0.298	0.132 (−0.005 to 0.268)	0.058	0.036 (0.080 to 0.153)	0.541
Model 4	Ref	0.082 (−0.038 to 0.203)	0.180	0.182 (0.048 to 0.316)	0.008	0.158 (0.035 to 0.281)	0.012
**(b) HIV and ART on post-FEV1/FVC**							
Model 1	Ref	0.005 (−0.012 to 0.022)	0.563	−0.010 (−0.027 to 0.008)	0.268	−0.026 (−0.040 to −0.012)	< 0.001
Model 2	Ref	−0.002 (−0.017 to 0.013)	0.791	−0.007 (−0.023 to 0.009)	0.400	−0.004 (−0.018 to 0.010)	0.566
Model 3	Ref	−0.002 (−0.018 to 0.014)	0.801	−0.007 (−0.024 to 0.010)	0.437	−0.004 (−0.019 to 0.011)	0.597
Model 4	Ref	−0.001 (−0.017 to 0.015)	0.892	−0.003 (−0.020 to 0.014)	0.739	0.006 (−0.010 to 0.022)	0.471

ART, antiretroviral therapy; FEV1, forced expiratory volume in 1 s; FVC, forced vital capacity.

Note: β-coefficient with 95% confidence interval. Model 1 unadjusted; Model 2 adjusted for age, sex and BSA; Model 3 adjusted for age, sex, BSA, current or ever smoking, employment and education (secondary school completed); Model 4 adjusted for age, sex, BSA, current or ever smoking, employment and education (secondary school completed), and tuberculosis.

Multicollinearity was tested using a correlation matrix and the variance inflation factor. In the sensitivity analysis, participants with less than three post-bronchodilator blows were excluded from the analysis. Participants with incomplete spirometry were no different from those with complete spirometry data, and as we had sufficient power, listwise deletion of participants without spirometry results was performed. All analyses were performed using Statistical Package for Social Sciences (SPSS) version 25 (SPSS, Chicago, IL, United States [US]). A two-sided *p*-value of < 0.05 was considered to be statistically significant.

## Results

Of the 548 participants enrolled, 531 participants (97%) were included in the analysis. Nine participants were excluded because of missing spirometry results and eight with poor-quality spirometry. Furthermore, eight participants had an insufficient post-bronchodilator test and, therefore, their results could not be used to assess asthma. The characteristics of the four different groups are discussed in this study, as shown in Table 1.

The majority were female participants (*n* = 329, 62%) with the mean age of 38 (s.d. 9.5) years. Almost all participants were black Africans (97.7%). HIV-positive participants were more often women and older compared with the HIV-negative participants (*P* < 0.01 for both comparisons). HIV-negative participants were more often current smokers than HIV-positive participants (37% vs. 14%, *P* < 0.001). Employment rate varied significantly between the groups, with the lowest employment rate reported for the HIV-negative group and the highest employment rate for the HIV+, ART1 group (33% vs 85%, *P* < 0.001). HIV-positive participants were much more likely to have had TB (30% vs 4%, *P* < 0.001). The median CD4 cell count was 470 among HIV-positive participants (IQR = 395).

The further analysis included 524 participants (96%) as 24 participants did not undergo a spirometry or had a spirometry that did not meet the quality criteria. These 24 participants had a similar demographic profile to those who completed the spirometry. The frequency of OLD was 8.3% (*n* = 44) in total (Table 2). For reversible airflow obstruction, it was 6.3% (*n* = 33) and for COPD, according to the LLN, it was 3.1% (*n* = 16) (Table 2).

Following adjustment for age, gender and BSA, no effect of HIV and ART on post-FEV1 was observed (Table 3a, model 2). Age was associated with a significantly lower FEV1 (β = −0.03 L, *P* < 0.001), and male gender and BSA were associated with a significantly higher FEV1 (β = 0.89 L and 0.36 L, respectively, *P* < 0.001). Additional adjustment for smoking, employment and education did not change the results (Table 3a, model 3). After further adjustment for TB (model 4), HIV-positive participants on first-line and on second-line ART had a significantly higher post-FEV1 compared with HIV-negative participants, whereas TB was associated with a lower FEV1 (β = −0.236 L, *P* < 0.001). No effect of HIV and ART on the post-FEV1/FVC was observed (Table 3b). Each variable of interest consisted of less than one percent missing. Therefore, multiple imputations were assumed to provide no benefit. In a sensitivity analysis, the above-described analyses were repeated whilst excluding 32 participants with only one or two reproducible post-bronchodilator flow volume curves. The magnitude and direction of the results were the same.

## Discussion

In this study, we sought to determine whether HIV and ART were independently associated with pulmonary function, taking TB into account. In this urban African population, the frequency of OLD was 8.3%. HIV and ART were associated with a significantly higher FEV1, but only following adjustment for TB, whilst TB was associated with a lower FEV1. This indicates a mediating effect of TB in the association of HIV and ART with pulmonary function as assessed with FEV1. No effect on the FEV1/FVC ratio was observed. The frequency of 3% for COPD in this study population is lower compared with that of previous studies from South Africa, with a prevalence rate of up to 22%.^34,35^

These studies, however, included older participants and more smokers. The reversible airflow obstruction frequency of 9% among HIV-positive participants in this study was slightly higher compared with the 7.3% found by Gingo et al.^36^ in the United States using the same definition. Limited available data regarding the prevalence of asthma complicates a comparison with earlier studies. In addition, most of these studies were performed with children and based on self-reported wheeze or previous diagnosis of asthma,^37,38^ or used a challenge test,^39^ both shown to diagnose asthma more frequently compared with using a bronchodilator test to measure reversible airflow obstruction.^40,41,42,43^ Asthma is typically diagnosed early in life and could have preceded inclusion in this study with a minimum age of 18 years. Consistent with the low frequency of OLD, this cohort has a surprisingly low frequency of respiratory complaints, as was previously described by Kummerow et al.^44^

Previous studies support an increased frequency of respiratory symptoms^2,10^ and incidence of OLDs among HIV-positive people using ART.^6,45,46,47^ Crothers et al.^6^ stated that HIV infection is an independent risk factor for COPD. However, these findings were based on the patient’s self-report and did not include women, a group that constitutes the largest proportion of the HIV population in SSA. Drummond et al.^7^ found that HIV independently contributes to an accelerated pulmonary function decline whilst taking into account respiratory infections. However, participants in that study were intravenous drug users, which is an independent risk factor for COPD.^2^ Both studies also reported a much higher smoking frequency compared with our study. As such, these studies are most likely not generalisable to SSA populations. A recent study in a rural African population found that pulmonary function was impaired in PLHIV compared with HIV-negative participants; however, the impairment may have resulted from co-infection with TB.^14^ The current study confirms this finding by showing a mediating effect of TB in the relationship between HIV, ART and lung function.

Studies have shown contradictory results on the effect of ART on pulmonary function. Crothers et al.^6^ concluded that the use of ART was associated with a lower risk of COPD. Contrasting these findings, two cross-sectional studies have shown that the use of ART is associated with an increase in the prevalence of COPD and a decline in the post-FEV1/FVC ratio.^2,10^ The current study showed no effect of using ART on the pulmonary function in PLHIV. These conflicting findings suggest that environmental and occupational factors, such as exposure to open fire, air pollution and the use of personal protection at dusty jobs, may vary between populations; however, it is also likely that methods of data collection, diagnosis and vital statistics are not directly comparable between studies.^48,49^

No effect of smoking on post-FEV1 and post-FEV1/FVC was observed, which may be because of irregular smoking habits depending on financial resources or lack of information on pack-years in current smokers, resulting in misclassification of smoking habits.^50^ An additional explanation could be that the study population is relatively young, and that the effect of smoking only appears later in life. Furthermore, HIV-positive participants might remember their medical history better, and social desirability bias could have occurred because of smoking stigma in HIV-positive people.

The finding that HIV is associated with an increase in FEV1 was surprising. There could be unmeasured variables associated with airway obstruction that were not considered in the current study, which could explain a lower FEV1 in the control group. However, most previously reported variables associated with pulmonary obstruction were included and tested. Furthermore, those on first- or second-line ART had a higher prevalence of TB. Therefore, the mediating effect of TB is larger in these groups, which was reflected by model 3. Another challenge lies in defining the clinical relevance of the study findings, as there is a small decline in FEV1 initially, and the impact of this decline on a patient depends on various factors.^48^ Because of the relatively young population in the current study, clinically relevant effects might not have occurred yet, but could still occur later in life.

Strengths of this study include the standardised data collection, including questionnaires and spirometry testing, and the use of an HIV-negative control group. To account for differences between the groups, the analyses were corrected for smoking, a known confounder in the relation between HIV and pulmonary function, and employment and education were added as a proxy for the SES. Furthermore, although participants were recruited from RCTs, we feel that they represent the general HIV-positive community.

Participants in the RCT were recruited at public HIV treatment clinics in the inner city of Johannesburg. Randomised controlled trials in this setting tend to attract the general population because of the conveniences in logistics (such as travel reimbursement, personal attention and shorter waiting times at the clinics).

A limitation of the study includes its cross-sectional design, limiting any causal inference. In order to assess the real burden of pulmonary impairment, a follow-up study would be more suitable. Moreover, it might be that the control group of this study is not completely representative of the general HIV-uninfected population. This study data showed that employment rates were much lower in the HIV-negative group. This might reflect that unemployed family members or friends were more eager to participate than employed relatives. This could have introduced selection bias despite adjustment for socio-demographic factors as there might have been unmeasured confounding. In addition, a history of TB was combined with a history of pneumonia, as we deemed this self-reported information not accurate enough to distinguish between both entities. This could have led to the overdiagnosis of TB.

In conclusion, the findings in this study suggest that HIV infection and ART use are not associated with a reduction in pulmonary function. However, HIV remains the main risk factor for acquiring TB and the data did show a reduced pulmonary function in relation to TB. This emphasises the important role of TB in this population. Future research studies should be targeted at eliminating TB, and an investigation of whether those living with HIV and a history of TB would benefit from screening tests to detect OLDs. In daily HIV care, healthcare providers should be aware that a history of TB can contribute to an impaired pulmonary function.
