# Corrigendum: The influence of HIV infection and antiretroviral treatment on pulmonary function in individuals in an urban setting in sub-Saharan Africa

**DOI:** 10.4102/sajhivmed.v23i1.1351

**Published:** 2022-05-20

**Authors:** Oda E. van den Berg, Erica J. Shaddock, Sarah L. Stacey, Charles Feldman, Roos E. Barth, Diederick E. Grobbee, Willem D.F. Venter, Kerstin Klipstein-Grobusch, Alinda G. Vos

**Affiliations:** 1 Julius Global Health, Julius Center for Health Sciences and Primary Care, University Medical Center Utrecht, Utrecht, the Netherlands; 2 Charlotte Maxeke Johannesburg Academic Hospital, Division of Pulmonology and Critical Care, School of Clinical Medicine, Faculty of Health Sciences, University of the Witwatersrand, Johannesburg, South Africa; 3 Department of Internal Medicine and Infectious Diseases, University Medical Center Utrecht, Utrecht, the Netherlands; 4 Ezintsha, University of the Witwatersrand, Johannesburg, South Africa; 5 Division of Epidemiology and Biostatistics, School of Public Health, Faculty of Health Sciences, University of the Witwatersrand, Johannesburg, South Africa

In the version of this article initially published, Van den Berg OE, Shaddock EJ, Stacey SL, et al. The influence of HIV infection and antiretroviral treatment on pulmonary function in individuals in an urban setting in sub-Saharan Africa. S Afr J HIV Med. 2021;22(1), a1312. https://doi.org/10.4102/sajhivmed.v22i1.1312, there was an error in affiliation 1. Instead of Julius Center for Health Sciences and Primary Care, University Medical Center Utrecht, Utrecht, the Netherlands, it should be Julius Global Health, Julius Center for Health Sciences and Primary Care, University Medical Center Utrecht, Utrecht, the Netherlands.

In addition, there was an error regarding the affiliation for Kerstin Klipstein-Grobusch. As well as having the affiliation Julius Global Health, Julius Center for Health Sciences and Primary Care, University Medical Center Utrecht, Utrecht, the Netherlands, he should also have Division of Epidemiology and Biostatistics, School of Public Health, Faculty of Health Sciences, University of the Witwatersrand, Johannesburg, South Africa.

The authors apologise for this error. The correction does not change the study’s findings of significance or overall interpretation of the study’s results or the scientific conclusions of the article in any way.

